# Provision of obstetrics and gynaecology services during the COVID‐19 pandemic: a survey of junior doctors in the UK National Health Service

**DOI:** 10.1111/1471-0528.16313

**Published:** 2020-05-27

**Authors:** MP Rimmer, BH Al Wattar, UKARCOG Members, Catriona Barlow, Naomi Black, Ciara Carpenter, Frances Conti‐Ramsden, John A W Dalton, Rhianna Davies, Rebecca Davies, Cheryl Dunlop, Elvena Guyett, Laura Jamison, Babu Karavadra, Lorraine Kasaven, Katherine Lattey, Emma Long, Caroline Macmahon, Kate Navaratnam, Simrit Nijjar, Stephen O'Brien, Obi Ojukwu, Laura Parnell, Olivia Raglan, Meera Ramcharn, Jenny Riches, Linden Jane Stocker, Siew Chee Wong, Charlotte Wyeth

**Affiliations:** ^1^ UK Audit and Research Collaborative in Obstetrics and Gynaecology London UK

**Keywords:** Coronavirus, coronavirus disease 2019, gynaecology, national health service, obstetrics, survey, women's health care

## Abstract

**Objective:**

The coronavirus disease 2019 (COVID‐19) pandemic is disrupting health services worldwide. We aimed to evaluate the provision of obstetrics and gynaecology services in the UK during the acute phase of the COVID‐19 pandemic.

**Design:**

Interview‐based national survey.

**Setting:**

Women's healthcare units in the National Health Service.

**Population:**

Junior doctors in obstetrics and gynaecology.

**Methods:**

Participants were interviewed by members of the UK Audit and Research in Obstetrics and Gynaecology trainees' collaborative between 28 March and 7 April 2020. We used a quantitative analysis for closed‐ended questions and a thematic framework analysis for open comments.

**Results:**

We received responses from 148/155 units (95%), most of the participants were in years 3–7 of training (121/148, 82%). Most completed specific training drills for managing obstetric and gynaecological emergencies in women with COVID‐19 (89/148, 60.1%) and two‐person donning and doffing of Personal Protective Equipment (PPE) (96/148, 64.9%). The majority of surveyed units implemented COVID‐19‐specific protocols (130/148, 87.8%), offered adequate PPE (135/148, 91.2%) and operated dedicated COVID‐19 emergency theatres (105/148, 70.8%). Most units reduced face‐to‐face antenatal clinics (117/148, 79.1%) and suspended elective gynaecology services (131/148, 88.5%). The 2‐week referral pathway for oncological gynaecology was not affected in half of the units (76/148, 51.4%), but half reported a planned reduction in oncology surgery (82/148, 55.4%).

**Conclusion:**

The provision of obstetrics and gynaecology services in the UK during the acute phase of the COVID‐19 pandemic seems to be in line with current guidelines, but strategic planning is needed to restore routine gynaecology services and ensure safe access to maternity care in the long term.

**Tweetable abstract:**

Provision of obstetrics and gynaecology services during the acute phase of COVID‐19 is in line with current guidelines, strategic planning is needed to restore routine services and ensure safe access to care in the long term.

## Tweetable abstract

Provision of obstetrics and gynaecology services during the acute phase of COVID‐19 is in line with current guidelines, strategic planning is needed to restore routine services and ensure safe access to care in the long term.

This paper includes Author Insights, a video abstract available at https://vimeo.com/rcog/authorinsights16313

## Graphical abstract

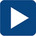

## Introduction

In less than 4 months, the emerging coronavirus disease 2019 (COVID‐19) pandemic has sent shock waves through the global health system, significantly disrupting care provision with high levels of morbidity and mortality worldwide.^1^ With more than three million cases and 200 000 deaths reported globally,^2^ many countries have implemented strict social distancing rules with full or partial lockdown. To meet the unprecedented demand for acute and intensive healthcare services, a rapid response is urgently needed to efficiently relocate resources, revise acute‐care pathways and maintain the safety and wellbeing of healthcare professionals.^3^

In times of crisis, much morbidity arises from poor access to health services and depletion of valuable resources.^4^, ^5^, ^6^ This is especially relevant to women's health where access to acute care is in continual demand.^7^ Recently, both the Ebola virus (2014/15)^8^ and the ‘swine flu’ influenza A virus (2009)^9^ epidemics had direct and indirect adverse effects on the wellbeing of women, leading to a significant increase in stillbirths and maternal mortality in affected communities.^10^, ^11^, ^12^, ^13^ Dealing with COVID‐19 is particularly challenging because of its asymptomatic presentation and the high risk of transmission to healthcare professionals.^14^ The worldwide shortage of personal protective equipment (PPE) has been a particular worry to healthcare professionals caring for affected patients at great personal risk.^13^ Poor logistical planning and the lack of rapid testing at the point of care have been a major worry for healthcare professionals in the UK National Health Service (NHS) facing this pandemic.^15^

Efficient assessment and feedback are key to refining the planned responses facing this pandemic and ensuring the safety of both the patients and their caring professionals. We aimed to provide a snapshot assessment of current service provision in obstetrics and gynaecology in the NHS by surveying junior doctors in all women's healthcare units in the UK.

## Methods

### Design

We conducted an interview‐based survey with junior doctors in obstetrics and gynaecology across all training units in the NHS. Participants were contacted via the UK trainees' collaborative for Audit and Research in Obstetrics and Gynaecology (UK ARCOG) network^16^ between 28 March and 7 April 2020. There was no funding for the study.

The interviews included a mixture of closed‐ and open‐ended questions to capture current practice and the adoption of guidance from the World Health Organization and Royal College of Obstetricians and Gynaecologists on the safe management of women with COVID‐19.^17^, ^18^, ^19^ The survey aimed to cover four main domains: availability of PPE, training specific to COVID‐19, changes in maternity care services and changes in gynaecology services (see Supplementary material, Appendix S1). We aimed to evaluate the participants' levels of anxiety and perception of confidence using simple Likert scales anchored between 1 (severe anxiety or no confidence) and 9 (no anxiety and very confident). We piloted the questions among members of the UK ARCOG core committee to ensure their face validity.

### Data collection

Data were collected by a nominated UK ARCOG representative at each Local Educational Training Board (LETB) using a standardised electronic data collection tool. Data were harmonised and merged centrally by the same moderator (MPR) and quality checked for any entry errors.

### Statistical analysis

We reported data using natural frequencies and percentages. Where relevant, we used Kruskal–Wallis test to compare medians of non‐parametric data with significance at *P* < 0.05.

We analysed open comments thematically using a framework analysis to understand the participants' experiences and perspectives.^20^ This was done in two steps, first we reviewed open comments and developed a list of emerging topics and salient themes. Afterwards, we coded all the submitted comments into the final framework. We reported on prominent themes arising from the framework narratively. All statistical analyses were performed using SPSS (version 22, SPSS Inc., Chicago, IL, USA) and Microsoft excel (version 16, Redmond, WA, USA).

### Core outcome sets and patient involvement

No core outcome sets exist for this area of research. We did not seek input from patients when developing this tool as it sought to seek responses from healthcare staff only.

## Results

We contacted all 155 training units in the UK across 16 LETBs. We received responses from 148/155 units (95%). The majority of participants were junior doctors in years 3–5 of their training (82/148, 55.4%), followed by years 6–7 (39/148, 25.9%), 1–2 (20/148, 13.5%) and non‐training trust grade doctors (7/148, 4.7%). We did not receive responses from the remaining seven units because of lack of engagement.

### COVID‐19 response

A majority of junior doctors reported having undergone specific training drills for dealing with obstetric and gynaecological emergencies in women with COVID‐19 (89/148, 60.1%) and two‐person donning and doffing of PPE (96/148, 64.9%), though only 80% had a face‐mask (FFP3 or equivalent) fit tested (119/148, 80.4%) (Table 1). Most participants expressed the need for more frequent training sessions on COVID‐19‐specific emergency drills and clearer e‐learning resources for specific skills such as two‐person donning and doffing of PPE (see Supplementary material, Table S1). Most participants felt confident about their unit's ability to deal with COVID‐19 appropriately (median 6, range 1–9), which was not different across the included LETBS (*P* = 0.15) (Table 1). General anxiety was on the higher side among participants (median 6, range 1–9), largely due to uncertainty about the future of the health service and the ability of the NHS to cope; this also was not different across the included LETBS (*P* = 0.08). In total, 62.8% of junior doctors felt that they were adequately supported by senior staff when dealing with this health crisis (Table 1). This was exemplified by increased presence of consultants on labour wards and regular communication from clinical managers (see Supplementary material, Table S1).

**Table 1:** Summary of junior doctors' responses on the provision of obstetrics and gynaecology services in the NHS response during the acute phase of the COVID‐19 pandemic

Question	Yes *n* (%)	No *n* (%)	Unsure *n* (%)
**Training and support**			
Have you carried out any COVID‐19 training drills for obstetric and gynaecological emergencies?	89 (60)	59 (40)	0 (0)
Have you been face‐fit tested for an FFP3 or equivalent mask?	119 (80.4)	29 (19.6)	0 (0)
Have you received training in two‐person donning and doffing of PPE?	96 (64.9)	52 (35.1)	0 (0)
Have you received specific training on the care for a woman with COVID‐19?	68 (45.9)	78 (52.7)	2 (1.4)
**Labour ward**			
Do you have a dedicated COVID‐19 operating theatre for obstetric emergencies?	105 (70.9)	42 (28.4)	1 (0.7)
Do you have access to PPE on the labour ward?	135 (91.2)	11 (7.4)	2 (1.4)
Has there been a clear protocol for management of suspected and confirmed COVID‐19 patients on the labour ward in your unit?	130 (87.8)	18 (12.2)	0 (0)
Have there been any planned changes in the number of inductions of labour and/or elective caesarean sections?	62 (41.9)	83 (56.1)	3 (2)
**Antenatal and postnatal care**			
Did you start providing antenatal care service over the phone/ videoconferencing?	105 (70.9)	33 (22.3)	10 (6.8)
Has there been a planned reduction in attendance to antenatal care?	117 (79.1)	19 (12.8)	12 (8.1)
Have there been any changes to antenatal screening pathways at your unit?	66 (44.6)	59 (39.9)	23 (15.5)
Has there been clear protocol to speed up inpatient discharge postnatally?	59 (39.9)	75 (50.7)	14 (9.5)
Are there dedicated bays or areas to care for women with suspected or confirmed COVID‐19?	116 (78.4)	21 (14.2)	11 (7.4)
**Benign gynaecology**			
Has the unit stopped all elective work including urogynaecology and fertility services?	131 (88.5)	12 (8.1)	5 (3.4)
Have there been protocols to avoid the use of emergency laparoscopy in women with suspected or confirmed COVID‐19?	65 (43.9)	73 (49.3)	10 (6.8)
Has there been protocol to offer medical management of miscarriage as a first‐line treatment?	87 (58.8)	51 (34.5)	10 (6.8)
Has there been protocol to offer medical management of confirmed ectopic pregnancy as a first‐line treatment?	42 (28.4)	89 (60.1)	17 (11.5)
**Oncology gynaecology**			
Have you changed your Two‐Week Wait referral pathway and/or services?	59 (39.9)	76 (51.4)	13 (8.8)
Have you reduced your oncology theatre lists?	82 (55.4)	53 (35.8)	13 (8.8)
Have you moved gynae‐oncology operating to a different site or trust?	37 (25)	102 (68.9)	9 (6.1)
Have you changed the ward where gynae‐oncology patients are cared for postoperatively?	39 (26.4)	100 (67.6)	9 (6.1)
**General**			
As a junior doctor in obstetrics and gynaecology, do you feel well supported facing this pandemic?	93 (62.8)	45 (30.4)	10 (6.8)
	**Median score**	**Range of scores**	
How do you rate your confidence in how your unit is managing this pandemic on a scale from 1 to 9? 1 being no confidence and 9 being very confident	6	1–9	
How do you rate your anxiety at present on a scale from 1 to 9? 1 being severe anxiety and 9 being no anxiety	6	1–9	

### Changes to obstetrics and gynaecology services

Most included units had developed departmental protocols on the management of pregnant women with suspected or confirmed COVID‐19 on the labour ward (130/148, 87.8%), though some participants reported that these protocols were changing rapidly, leading to confusion and unclear interpretation by staff members (see Supplementary material, Table S1). Most labour wards offered adequate access to PPE (135/148, 91.2%) and operated dedicated COVID‐19 theatres for obstetric emergencies (105/148, 70.8%). There were some shortfalls in elective obstetric care planning (caesarean section and induction of labour) with just over one‐third of units adapting their practice to the current crisis (62/148, 41.9%). Many participants commented that reasons for induction of labour were scrutinised by senior staff to reduce inpatient admissions as well as promoting outpatient induction of labour using intracervical catheters. Some comments alluded to the suspension of elective caesarean sections for maternal requests (see Supplementary material, Table S1). About one‐third of units implemented protocols to reduce inpatient stay in the postpartum period (59/148, 39.9%).

Most units altered the provision of antenatal care by reducing face‐to‐face clinics (117/148, 79.1%), offering more telephone or virtual consultations (105/148, 70.9%) and identifying dedicated clinic areas for pregnant women with suspected or confirmed COVID‐19 (116/148, 78.4%). More than one‐third altered their screening pathways to reduce in‐hospital attendance (66/148, 44.6%) with some participants reporting the use of fasting blood sugar and HbA1c instead of oral glucose tolerance test to reduce in‐hospital stay and reduction of the frequency of serial growth scans as common measures (see Supplementary material, Table S1).

At the time of this survey, the majority of units had suspended their elective gynaecology services including fertility and urogynaecology (131/148, 88.5%) with some units operating virtual clinics (see Supplementary material, Table S1). About two‐thirds of surveyed units started offering medical management as the first line of treatment for miscarriage (87/148, 58.8%) and ectopic pregnancies (42/148, 28.4%) to reduce inpatient stays in acute gynaecology wards. Many participants expressed concerns for the lack of clarity on using laparoscopy in COVID‐19 patients with only two‐thirds of units adopting laparotomy as first‐line surgical approach in such women (65/148, 44%).

Around half of the surveyed units did not change the timelines of the gynaecology oncology 2‐week referral pathway (76/148, 51.4%), but half reported a planned reduction in oncology operating lists (82/148, 55.4%). Only a minority moved oncology surgery to a COVID‐19‐free site such as the premises of non‐NHS private hospitals (37/148, 25%) as well as postoperative wards (39/148, 26.4%).

## Discussion

### Main findings

Our findings offer a rapid comprehensive overview of the provision of obstetrics and gynaecology services in the NHS in response to the COVID‐19 pandemic.

Overall, the majority of units seem to be following national guidance during the acute phase of the crisis but variation in practice and misinterpretation of guidance were expressed by our participants, especially where limited evidence is available to inform practice such as the emergency gynaecology care.^17^, ^21^ Most surveyed units were offering adequate PPE and revising care pathways to safely manage women with COVID‐19 with an apparent reliance on e‐learning to promote training and education on the safe use of PPE.^13^, ^22^ We acknowledge that little time has elapsed to allow adequate synthesis and implementation of evidence‐based guidelines, but measures to protect healthcare workers are crucial to sustain the workforce, respond to the rising demand for acute care and avoid the collapse of the healthcare system.^23^, ^24^ Although there was an overall sense of anxiety among junior doctors, the majority felt well supported by senior colleagues when caring for women with COVID‐19. Many trainees expressed the need for more practical training and the desire to be more involved in the planning of future service provision.

### Strengths and limitations of findings

Our findings are limited by the scope of the interview questions and the limited representation of each unit (one participant per unit). A detailed analysis is likely to yield more information on the current practice and to identify areas for improvements in women's healthcare provision. This is especially relevant for topics such as the availability of PPE, which could change daily across the surveyed units. Still, our findings are comprehensive and contemporary to evaluate the COVID‐19 response across all women's healthcare units.

### Interpretation and implications for practice

The COVID‐19 crisis is likely to have both short‐term and long‐term impacts on women's healthcare services worldwide. Our findings suggest an overall good initial response in the NHS during the acute phase of this pandemic in line with current evidence‐based health policies and clinical guidelines. Still, some alarming features emerged, such as the suspension of elective gynaecology, the deferring of antenatal screening tests and the reduction of oncology operating capacity. Prolonged suspension of these essential services is likely to increase morbidity in the long term.

Strategic planning is, therefore, required to mitigate the risk of the adverse outcomes to women's health seen during similar crises.^4^, ^7^ As we flatten the infection curve awaiting an effective vaccine, regular evaluation of care provision is essential to optimise our response and ensure women can safely access the care they need.^25^ This is especially relevant to essential, though non‐acute, services such as contraception, termination of pregnancy and cancer treatment.

Rapid testing and triaging systems for COVID‐19 patients are urgently needed, especially beyond the acute phase, to minimise the risk of exposure of unaffected women and their healthcare professionals. This is likely to become a daily practice as we transform to the ‘new normal’ over the next 18 months until reliable treatments and vaccines are developed.

Using innovative interventions to reduce inpatient stay in hospital has been pivotal in managing this crisis powered by the rapid uptake of telemedicine technology.^26^ Such interventions are likely to gain momentum worldwide giving rise to a new healthcare system where women can undergo diagnostic testing and be followed up by their caring clinicians at distance.^26^, ^27^ It is important, however, to rationalise their use because not all aspects of antenatal and postnatal care can be delivered using telemedicine. Reducing face‐to‐face antenatal appointments could increase pregnancy complications in high‐risk groups such as victims of domestic violence, deprived populations with limited access to advanced technology and those with mental health issues.^28^, ^29^

As with every new endeavour, efficient data collection and analysis are essential elements to advise our response to this global pandemic. Our findings shed some light on the impact of COVID‐19 on obstetrics and gynaecology healthcare services in the UK, but more stringent long‐term data collection and sharing are needed to fully evaluate the impact of this pandemic on women's health outcomes and to coordinate a global response to eliminate it.

## Conclusion

The provision of obstetrics and gynaecology services in the UK during the acute phase of the COVID‐19 pandemic seems to be in line with current guidelines, but strategic planning is needed to restore routine gynaecology services and ensure safe access to maternity care in the long term.

### Disclosure of interests

None declared. Completed disclosure of interests forms are available to view online as supporting information.

### Contribution to authorship

BHAW and MPR conceived the idea, wrote the protocol, analysed data and wrote the final manuscript. All other co‐authors actively helped in data collection and drafting of the final manuscript.

### Details of ethics approval

Not required.

### Funding

None.

## Supporting information


**Table S1.** Thematic analysis of participants’ open comments on the provision of obstetrics and gynaecology care in the NHS during the acute phase of the COVID‐19 pandemic.

**Appendix S1.** Questionnaire on the provision of obstetrics and gynaecology services in the NHS response during the acute phase of the COVID‐19 pandemic.

**Video S1.** Author Insights.

Supplementary Material

Supplementary Material

Supplementary Material

Supplementary Material

Supplementary Material

Supplementary Material

Supplementary Material

Supplementary Material

Supplementary Material

Supplementary Material

Supplementary Material

Supplementary Material

Supplementary Material

Supplementary Material

Supplementary Material

Supplementary Material

Supplementary Material

Supplementary Material

Supplementary Material

Supplementary Material

Supplementary Material

Supplementary Material

Supplementary Material

Supplementary Material

Supplementary Material

Supplementary Material

Supplementary Material

Supplementary Material
