# Supplementary material for: Provision of obstetrics and gynaecology services during the COVID‐19 pandemic: a survey of junior doctors in the UK National Health Service
Source: BJOG. 2020 May 27;127(9):1123–8. doi: 10.1111/1471-0528.16313 (PMC7283977; doi:10.1111/1471-0528.16313)
Supplement: Supplementary file 1 — Table S1. Thematic analysis of participants’ open comments on the provision of obstetrics and gynaecology care in the NHS during the acute phase of the COVID‐19 pandemic. [file BJO-127-1123-s029.pdf]

Table S1. Thematic analysis of participants open comments on the provision of obstetrics and gynaecology care in the NHS during the acute phase of the COVID-19 pandemic

Theme	Comments	
**CVOID training drills for obstetric and gynaecological emergencies**	Drills have occurred, but infrequently, and not publicised so not attended by all.<br><br>I think that there has been one drill run in the department which involved a small number of people. We have not yet been given feedback on it.<br><br>I know that one has taken place, but it was only for staff on labour ward that day.<br><br>There has been one drill run on the labour ward (category 2 caesarean section for a patient with no symptoms that was run as if she was COVID-positive). There are more drills planned for this week.<br><br>Just labour ward.	We had one drill but I was not a participant due to night shift work. No plans for further drills yet.<br><br>Other team members have, drills have occurred on occasional shifts.<br><br>Scheduled to take place, but lead for these drills is now self-isolating.<br><br>We have done a multi-disciplinary team drill (involved midwives, obstetricians, theatres, anaesthetists and paediatricians) of a patient; from arrival to emergency caesarean section under general anaesthetic. All information passed to staff not present and further drill in pipeline.
**Face fit tested for a FFP3 or equivalent mask**	Pregnant, non-clinical, other trainees have.<br><br>Prioritised by those first on call. Those who have failed are not expected to see suspected/confirmed cases.<br><br>Drop in sessions available for staff.	We have regular slots which we can book into.<br><br>Yes, however I failed the test and no alternative provided.
**Training donning and doffing of PPE**	There has been training at a local teaching session, although I wasn't personally present, as I was on nights.<br><br>An oral presentation in a drop-in session.<br><br>Email with video link.	There have been daily demonstrations before the start of the elective section list and all are welcome to attend.<br><br>Pictures on COVID news board.
**Specific training on the care for a woman with COVID**	Flow charts to assess patients in the maternity assessment unit have been disseminated.<br><br>Daily updates, new plan of care to be issued.<br><br>Local guidelines have been implemented and are regularly updated.	We have been advised to arrange a face mask fitting when on duty. We have been sent a critical care online training module.<br><br>Documents from Royal College of Obstetricians and Obstetricians/Royal College of Surgeons shared amongst specialty doctors.<br><br>Protocol through email.
**COVID specific theatres for obstetric emergencies**	Not specifically COVID/non-COVID theatres. I anticipate that some theatres will become Intensive care unit bays.<br><br>COVID positive patients to be operated on in main theatres; one theatre has been assigned for all COVID cases.	As so many positive patients we are no longer segregating theatres. It takes 15 minutes to let air circulate and for everything to be cleaned with appropriate wipes.<br><br>Shared theatre with other surgical specialties.

**Staff access to PPE on labour ward**	I haven't been fit tested yet.<br><br>Yes, it's available at the main desk.<br><br>I have not seen any but told it is available.<br><br>Box of FFP3 masks to be used only if aerosol generating procedure.<br><br>Only for theatre and outside the COVID - allocated delivery room.	We are only meant to use full personal protective equipment in second stage and theatre cases.<br><br>Even if confirmed or suspected case for a simple patient review, we are told to use a simple surgical mask, gloves and apron (no sleeve protection).<br><br>Protocol is not clear and it is interpreted differently by different staff members.
**Local guidance for management of suspected and confirmed COVID patients on labour ward**	There is a protocol regarding personal protective equipment use, no clear guidance regarding actual management, that is based on clinical scenario and circumstances.<br><br>Yes - very clear from outset. Even before we had COVID patients.<br><br>Constantly changing- sometimes difficult to keep up with multiple changes in a day theatre.	Rapidly changes reflecting the evolving guidance from government on personal protective equipment.<br><br>Multiple documents defining pathways, making diagnoses, how these should be managed in the unit and specific advice regarding management to avoid need for general anaesthetic sections in COVID mothers, particularly emphasising early consultant obstetric involvement and as a result there is now a consultant resident overnight as well as during the day.
**Provision of induction of labour and/or elective caesarean sections**	Rationalised throughout the week and weekend to try and keep workload evenly spread.<br><br>Numbers have not changed but protocol for induction has been changed to reduce time spent in hospital by women. Now using Prostin straight away if possible.<br><br>Now offering outpatient induction of labour for low risk women >37 weeks geststion.<br><br>Revision of all decisions by senior staff and modifications if possible.<br><br>Stopped induction of labour for maternal request/social reasons.<br><br>Only two inductions of labour/day (usually four).	All inductions and elective caesarean sections vetted by consultants.<br><br>No overbooking of inductions, increased capacity to three all day elective c-section lists rather than half day lists per week.<br><br>Reduced elective c-section for maternal request so the majority have a true medical indication.<br><br>Yes, we now do daily c-section lists.<br><br>Caesarean section lists reduced.<br><br>No planned changes to inductions.<br>Delivering elective caesarean sections in main theatres.
**Provision of antenatal care**	Started virtual antenatal clinics on case by case basis. Still offering face to face too.<br><br>Virtual clinics planned but not yet in action.<br><br>Consultants triaging who it to attend for face-to-face.<br><br>Started in gynaecology, filtering to ante-natal clinic, with routine/non-urgent appointments.	Patients not allowed to attend with partners.<br><br>Scan pathways changed to reduce number of growth scans where possible.<br><br>No longer doing oral glucose tolerance test to screen gestational diabetes, only fasting blood glucose.<br><br>No firm plans as yet but discussion is being had. All scans delayed until patient symptom free.

	Seeing asymptomatic patients at beginning of clinic, i.e. ones who need scans, then seeing symptomatic patients at end of clinic.	Body mass index threshold for glucose tolerance testing increased from increased from 30 to 35.
**Provision of postnatal care**	Encouraged early discharge but not formally set by protocol.<br><br>Decision for senior review to facilitate timely discharge.<br><br>Consultant leads the postnatal ward round.	But discharges have been expedited by a consultant ward round of postnatal patients.<br><br>Six hours discharge encouraged if suitable, aim to discharge majority of caesarean section on day 1.
**Care for women with suspected or confirmed COVID?**	Certain rooms been dedicated on the delivery suite, and a COVID medical assessment unit in the birthing centre.<br><br>Two dedicated rooms on labour ward, including one for scanning antenatal patients with suspected/confirmed COVID.	At the moment we have to use a room on delivery suite to provide care for the woman in antenatal/intrapartum/postnatal period.<br><br>A ward has been set aside to be used for COVID patients as well as two labouring rooms.
**Provision of elective gynaecology services service**	Only via telephone consultations.<br><br>Some gynae clinics going ahead but via telephone.	Did not stop completely, changed to phone consultation.
**Provision of emergency surgery in women with suspected or confirmed COVID**	Following Royal college of Obstetricians and Gynaecologists/British Society of Gynaecology Endoscopy guidance.<br><br>In discussions since surgical and British Society of Gynaecology Endoscopy guidelines produced.	Only informed on Friday to avoid laparoscopy but how to do this not confirmed.<br><br>In keeping with Royal college of Obstetricians and Gynaecologists/British Society of Gynaecology Endoscopy advice.
**Medical management of miscarriage and ectopic pregnancy**	No protocol for this as yet.<br><br>Manual vacuum aspiration emphasised as elective evacuation of retained products of conception will not be possible.<br><br>No protocol, but avoiding surgical evacuation of retained products of conception as much as possible.<br><br>No change in the usual protocol yet.	This is standard in our unit anyway.<br><br>Consultants keen to avoid surgery for ectopic but no set protocol to govern this as yet.<br><br>No plan as yet, it is currently in discussion.<br><br>No change in the usual protocol yet.
**Provision of oncology gynaecology services**	Each referral screened for need<br>Rapid access/post-menopausal clinic which are still running. I did some last week and patients did attend.<br><br>Telephone clinic, encourage Pipelle biopsy at one stop clinic if possible.<br><br>Telephone triage, 1 stop clinics still running.<br><br>Urgent cases being reviewed at one stop clinics by consultants.<br><br>One stop and post-menopausal bleeding clinics running as usual.	New oncology pathway has been formulated (I have not read it yet); certain cancers are not being operated on at all anymore.<br><br>Operating in different theatres as previous gynae theatres are now intensive care unit overflow.<br><br>From 30/03 all elective procedures (including oncology patients) have stopped.<br><br>Has moved from gynae theatre suite to general theatres (gynae theatre suite has been converted to intensive care unit space).

	All elective procedures (including oncology patients) have stopped.<br><br>We are trying to follow British gynaecological cancer society guidance so more reliance on chemotherapy and radiotherapy in the first instance. Large debulking surgery with need for intensive care unit has been scaled back and some surgeries have become far less ambitious with a greater acceptance of suboptimal debulking.	In progress, location not yet confirmed, but after this week there will be no gynae oncology operating in our site.<br><br>Ward has changed 3 times in the past fortnight.<br><br>Same ward in use but red and green zones foe COVID and non-COVID patients.<br><br>There is a red and green area in gynaecology ward for suspected or confirmed COVID and non-COVID patient.
**Support for junior doctors**	Yes, in terms of personal protective equipment, no as lack of advice about how things will change, redeployment etc. Advice is changing daily regarding personal protective equipment with no evidence or explanation for why it keeps changing.<br><br>Very supported by seniors.<br><br>Difficult to answer with yes or no. The trust and school are making some effort with lots of daily emails to staff. However, there has been no departmental teachings, presentations or formal discussion regarding the changes in service and junior doctors' support. The plans seem vague at times.<br><br>Junior doctors do not seem to be involved in the discussion regarding rota, service changes or potential redeployment. Also, no recognition of the potential impact on the emotional and mental wellbeing of trainees.<br><br>The rota is an issue for juniors, management have introduced more onsite on calls (out of hours).<br><br>In labour ward the charge midwives are keeping us right with protocol and personal protective equipment. As juniors we aren't involved in many of the training sessions, but I have been assured that the charge midwives will keep us right.<br><br>Daily update from the clinical director.<br><br>Management visible and on shop floor.	Consultants have arranged to cover the wards and antenatal assessment. We are very well supported during the obstetric clinical activities that are ongoing. However due to the nature of COVID we will have to forego a lot of training opportunities (virtually no gynae clinics or theatre, reduced ante-natal clinic, no scan training).<br><br>A general feeling of chaos and the unknown.<br><br>I feel well supported by my unit support. I wish I could say the same for higher levels of authority and health care planners/ decision makers.<br><br>Response from the unit seem to be very slow. Standard operating procedures are not communicated to us quickly which makes it confusing when we're on call.<br><br>I think the right steps are being taken however the specific communication for maternity should be clearer e.g. send a daily update email when lots of changes are happening.<br><br>I do not feel particularly well-supported; however, I think this reflects the lack of understanding and experience with the disease, rather than lack of effort or good intent. Talking to friends who are trainees elsewhere in the UK, I do not feel that my unit is significantly worse than others.<br>I am aware that the unit is learning and evolving daily.<br><br>General anxious feeling amongst trainees.
