# Supplementary material for: Provision of obstetrics and gynaecology services during the COVID‐19 pandemic: a survey of junior doctors in the UK National Health Service
Source: BJOG. 2020 May 27;127(9):1123–8. doi: 10.1111/1471-0528.16313 (PMC7283977; doi:10.1111/1471-0528.16313)
Supplement: Supplementary file 2 — Appendix S1. Questionnaire on the provision of obstetrics and gynaecology services in the NHS response during the acute phase of the COVID‐19 pandemic. [file BJO-127-1123-s028.pdf]

Appendix S1. Questionnaire on the provision of obstetrics and gynaecology services in the NHS response during the acute phase of the COVID-19 pandemic**.**

**Question**	Yes / No	Comments
**Training and support**		
Have you carried out any CVOID training drills for obstetric and gynaecological emergencies?		
Have you been face fit tested for a FFP3 or equivalent mask?		
Have you received training in 2 people donning and doffing of PPE?		
Have you received specific training on the care for a woman with COVID?		
**Labour Ward**		
Do you have a dedicated COVID operating theatre for obstetric emergencies ?		
Do you have access to PPE on labour ward?		
Has there been a clear protocol for management of suspected and confirmed COVID patients on labour ward in your unit?		
Have there been any planned changes in the number of induction of labour and/or elective caesarean sections?		
**Antenatal and postnatal Care**		
Did you start providing ANC service over the phone/videoconferencing?		
Has there been a planned reduction in attendance to ANC?		
Have there been any changes to antenatal screening pathways at your unit?		
Has there been clear protocol to speed up in-patient discharge postnatally?		
Are there dedicated bays or areas to care for women with suspected or confirmed COVID?		
**Benign gynaecology**		
Has the unit stopped all elective work including urogynaecology and fertility service?		

Has there been protocols to avoid the use of emergency laparoscopy in women with suspected or confirmed COVID?		
Has there been protocol to offer medical management of miscarriage as a 1st line treatment developed?		
Has there been protocol to offer medical management of confirmed ectopic pregnancy as a 1st line treatment developed?		
**Oncology gynaecology**		
Have you changed your Two Week Wait referral pathway and/or services?		
Have you reduced your oncology theatre lists?		
Have you moved Gynae-Oncology operating to a different site or trust?		
Have you changed the ward where Gynae-oncology patients are cared for post op?		
**General**		
As a junior doctor in O+G, do you feel well supported facing this pandemic?		
How do you rate your confidence in how your unit is managing this pandemic on a scale from 1-9?<br>1 being no confidence and 9 being very confident.		
How do you rate your anxiety at present on a scale from 1-9?<br>1 being severe anxiety and 9 being no anxiety.		
